# Cholesterol efflux is LXRα isoform-dependent in human macrophages

**DOI:** 10.1186/1471-2261-14-80

**Published:** 2014-07-04

**Authors:** A Zhi Sha Ma, Zhi Yuan Song, Qian Zhang

**Affiliations:** 1 Department of Cardiology, Southwest Hospital, The Third Military Medical University, Chongqing, China; 2 Department of Cardiology, The fifth hospital of Chinese PLA, Yinchuan, China

**Keywords:** Reverse cholesterol transport, Liver X receptor, siRNA, ABC transporter, Atherosclerosis

## Abstract

**Background:**

The nuclear receptor liver X receptor (LXR) has two isoforms: LXRα and LXRβ. LXR activation promotes cholesterol efflux in macrophages, but the relative importance of each LXR isoform in mediating cholesterol efflux remains elusive.

**Methods:**

We evaluated the ability of different doses of LXRs agonist T0901317 to affect cholesterol efflux in human macrophages and its relationship with mRNA and protein levels of several well-characterized proteins involved in cholesterol efflux, including ABCA1, ABCG1, SR-BI, LXRβ and LXRα, using quantitative real-time PCR, Western blotting, and siRNA techniques.

**Results:**

Here we show that LXRα rather than LXRβ sustains baseline cholesterol efflux in human blood-derived macrophages. Treatment of human macrophages with a non-isoform-specific LXR agonist T0901317 substantially increased HDL- and apoA-I-mediated cholesterol efflux, which was associated with increased mRNA and protein expression levels of ABCA1, ABCG1, SR-BI, LXRα and LXRβ. The siRNA- mediated silencing of LXRα, but not LXRβ significantly reduced the protein levels of ABCA1,ABCG1, and SR-BI as wellas HDL- and ApoA1-mediated cholesterol in human macrophages.

**Conclusions:**

These findings imply that LXRα- rather than LXRβ- specific agonists may promote reverse cholesterol transport in humans.

## Background

Atherosclerosis is characterized by the presence of cholesterol-laden macrophages (foam cells) within the arterial wall [1,2]. The formation of foam cells is a result of disrupted balance between cholesterol uptake and efflux in macrophages. Macrophage cholesterol efflux is predominantly mediated by ATP-binding cassette (ABC) transporters A1 (ABCA1), ABCG1, and scavenger receptor class B type I (SR-BI) [3]. It is the initial step of reverse cholesterol transport (RCT), a process that removes excess cholesterol from peripheral tissues/cells including macrophages to circulating high density lipoproteins (HDLs) for fecal disposal via the hepatobiliary route [4]. Liver X receptors (LXRs) are nuclear receptors that function as cholesterol sensors and regulate transcription of a set of genes associated with cholesterol absorption, transport, efflux and excretion, thus playing a pivotal role in cholesterol homeostasis in vivo [5]. There are two LXR isoforms, LXRα and LXRβ. Each of them forms a heterodimer with a retinoid X receptor (RXR) to activate target gene expression by 3 binding to LXR response elements (LXREs) located in the promoter 3 regions of the target genes [6]. LXRα and LXRβ are similar in structure, ligand-binding domains (LBDs), and DNA-binding domains (DBDs), but their nuclear retention and localization as well as functions display some differences [7]. The two isoforms may have evolved from one ancestor. Pufferfish has only one LXRα, which is more closely related to mammalian LXR genes by sequence similarity, yet the pattern of tissue expression more closely resembles mammalian LXRβ genes in its ubiquity of expression [8]. The sequence data suggests that the two LXR isoforms are likely duplicated from a single ancestor LXR gene, and this duplication is concurrent with the evolution of mammals [9]. In mammals, LXRα is abundantly expressed in the liver, adipose tissue, small intestine, kidneys and macrophages, whereas the LXRβ isoform is ubiquitously expressed [10]. T0901317 is a general agonist for both LXR isoforms [11]. It has been shown that the activation of LXRs by T0901317 facilitates cholesterol efflux in macrophages and inhibits atherosclerosis in animal models [12,13]. However, the relative importance of each LXR isoform in mediating cholesterol efflux in human macrophages remains elusive. In this study, we demonstrate that the baseline cholesterol efflux in human blood-derived macrophages depends on LXRα, but not LXRβ, implying a potential role of LXRα- specific activation in enhancing reverse cholesterol transport in humans.

## Methods

### Materials

LXRs agonist T0901317 was from Sigma, USA. Total RNA extraction reagent RNAiso Plus, PrimeScript RT reagent kit, and SYBR-Green PCR kit were obtained from Takara (Japan). Immunoblot reagents were purchased from the Beyotime Institute of Biotechnology (China). LXRα siRNA and LXRβ siRNA were synthesized by Shanghai Genechem (Shanghai, China). All other chemicals were of the best grade available from commercial sources.

### Cell culture

Human peripheral blood monocytes were isolated from blood samples of 3 healthy volunteers using Ficoll/Hypaque gradient centrifugation. The pooled monocytes were incubated in DMEM supplemented with 10% autologous serum for 10 days so that they would differentiate into macrophages. Written informed consent was obtained from all subjects for participation in the study, and the protocol was approved by the ethics committee of Southwest Hospital.

### Cellular cholesterol efflux assays

Human macrophages were cultured as indicated above. Macrophages were then labeled with [^3^H]-cholesterol (0.3 μCi/mL) in serum-free DMEM containing 50 μg/mL ox-LDL and 0.2% bovine serum albumin (BSA) for 24 h. The cells were washed twice with phosphate-buffered saline (PBS) and incubated in 25 mL of DMEM containing 0.2% BSA with or without LXRs agonist T0901317 at 5 or 10 μmol/L for 48 h. The media were then replaced with DMEM containing 0.2% BSA in the presence of lipid-free apoA-I (10 μg/mL) or HDL (50 μg/mL) for 24 h. Efflux media were collected and centrifuged to remove floating cells. Monolayer cells were washed twice with PBS, and cellular lipids were extracted using isopropanol. The radioactivity in media and cell-associated [^3^H]-cholesterol was then measured using a liquid scintillation counter. The percent efflux was calculated with the following equation: [total medium counts/ (total cellular counts + total medium counts)] × 100%.

### RNA isolation and quantitative realtime PCR analysis

Total RNAs were extracted using RNAiso Plus reagents according to manufacturer’s instructions. PCR primers were synthesized by Shanghai Sangon (Shanghai, China) and the primer sequences used were as follows: ABCA1: forward primer: 5’-AAG CCA AGC ATC TTC AGT TC-3’, reverse primer: 5’-CCA TAC AGC AAG AGC AGA AGG-3’; ABCG1: forward primer: 5’-ATA CAG GGG AAA GGT CTC CAA T-3’, reverse primer: 5’-CCC CCG AGG TCT CTC TTA TAG T-3’; SR-BI: forward primer: 5’-AGG GAT AGG GTT GGA GTC AGC-3’, reverse primer: 5’-CGT TGT AAT GGA AGC CAG AGG-3’; LXRα: forward primer: 5’-AGG CCG GTG CTG AGT ATG TC-3’, reverse primer: 5’-GGG CTC CAT AAA GTC ACC AA −3’; LXRβ: forward primer: 5’-TGT CGT GTG CTC AGT ATG TG-3’, reverse primer: 5’-AGC CGC CAT ATA GTC ACT GT-3’; and GAPDH: forward primer: 5’-AGG CCG GTG CTG AGT ATG TC-3’, reverse primer: 5’-TGC CTG CTT CAC CAC CTT CT-3’. Real-time quantitative PCR was performed with SYBR® Premix Ex Taq™ II on a Bio-Rad LightCycler with an iQ3.1 realtime PCR system. Melt curve analysis of all real-time PCR products was used to produce a single DNA duplex. Quantitative measurements were obtained using the ∆∆Ct method. GAPDH was used as an invariant internal control.

### Western blot analysis

Cells were harvested and protein extracts prepared in accordance with the manufacturer’s instructions. Immunoblot analysis [12% SDS-PAGE; 30 μg proteins per lane] was then performed using rabbit anti-ABCA1, anti-ABCG1, anti-SR-BI, anti- LXRα, anti-LXRβ and anti-GAPDH antibodies (Abcam, USA). Proteins were visualized using Enhanced Chemiluminescence.

### Screening for effective LXR siRNA fragments

SiRNAs specific for human LXRα and LXRβ and the nonsilencing (control) siRNAs were synthesized by Shanghai Genechem (Shanghai, China). Human macrophages (1 × 10^6 cells/well) were transfected with each siRNA using Lipofectamine2000 (Invitrogen). Following 48 h transfection, the second siRNA fragment (5’-AAC TCA ATG ATG CTG AGT T-3’, LXRα-siRNA) targeting LXRα suppressed LXRα expression by 70%, and the third siRNA fragment (5’-ATG TCA CTG ATT CTG AGT AA-3’, LXRβ- siRNA) targeting LXRβ suppressed LXRβ expression by 75% according to realtime PCR results.

### LXR siRNA transfection and Western blot analysis

Human macrophages were grown in culture flasks at a density of 1 × 10^7/mL for 12 h, washed twice with PBS, and then incubated in DMEM containing 10% autologous human serum. The non-targeting control siRNA, LXRα siRNA and LXRβ siRNA were added to the culture flasks separately, and cultured for 96 h. Cells were then harvested and protein extracts prepared in accordance with the manufacturer’s instructions. The proteins were then subjected to immunoblot analysis [12% SDS-PAGE; 60 μg protein per lane] using a rabbit anti-ABCA1, anti-ABCG1, anti-SR-BI, anti- LXRα, anti-LXRβ or anti-GAPDH (Abcam, USA)-specific antibody. Proteins were visualized using Enhanced Chemiluminescence reagents.

### Cholesterol efflux assays

Human macrophages were cultured as indicated above. Human macrophages were then labeled with [^3^H]-cholesterol (0.3 μCi/mL) in serum-free DMEM containing 50 μg/mL ox-LDL and 0.2% BSA for 24 h. The cells were washed twice with PBS, cultured in DMEM containing 0.2% BSA, then treated with siRNAs as described above. Seventy-two hours post-siRNA treatment, the media were replaced with DMEM containing 0.2% BSA and lipid-free apoA-I (10 μg/mL) or HDL (50 μg/mL) for cholesterol efflux assays as described above.

### Statistical analysis

Data are expressed as Mean ± Standard Error of the Mean (SEM). Results were analyzed using one-way ANOVA with SPSS 13.0 software. P < 0.05 was considered statistically significant.

## Results

### LXRs agonist T0901317 enhances cholesterol efflux in human macrophages

**Figure 1:**
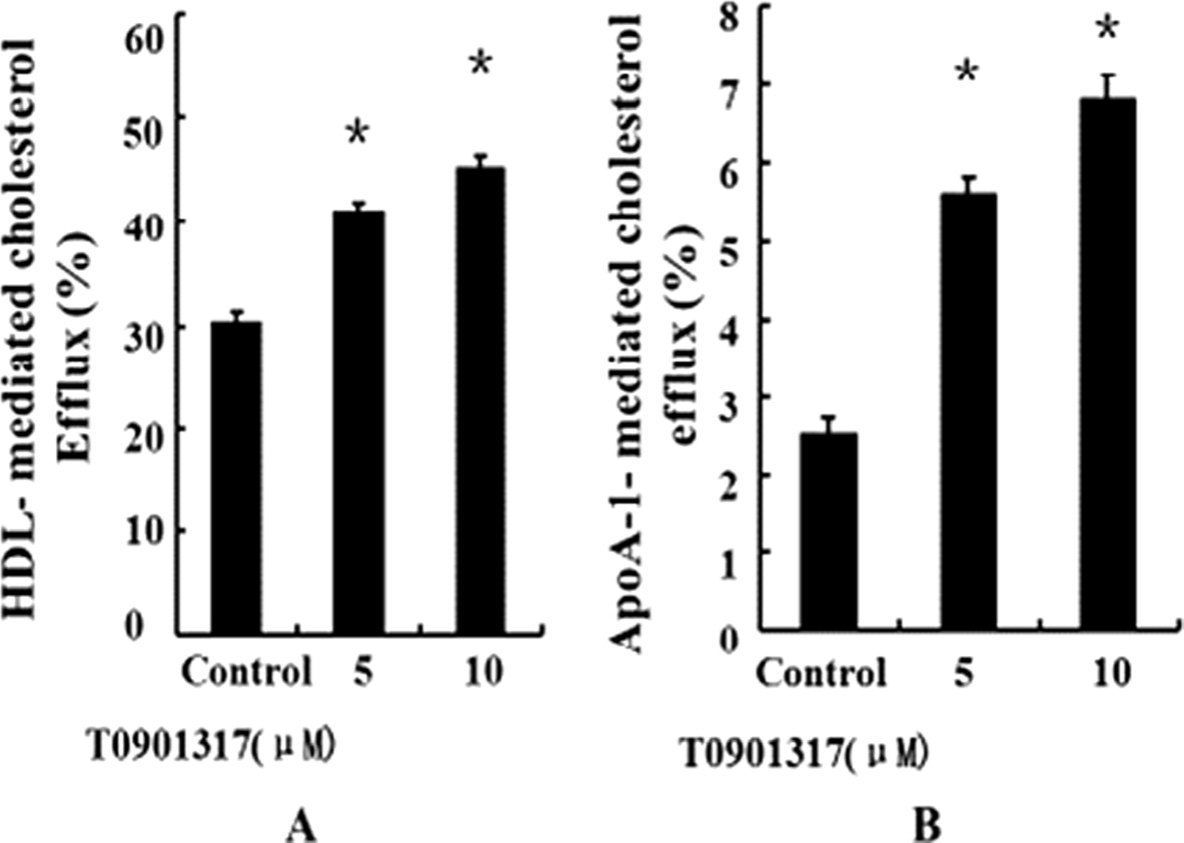
**LXRs agonist T0901317 enhances cholesterol efflux from human macrophages.** Following 24 h of labeling with [^3^H]-cholesterol, human macrophages were treated with T0901317 or the vehicle for 48 h. Cells were then washed with PBS, and assayed for apoA-I- **(A)** or HDL- **(B)** mediated cholesterol efflux as described under the Methods. Data in each group were obtained from triplicate flasks. Data are presented as Mean ± SEM, *P < 0.05 (vs control).

To determine if LXRs agonist T0901317 promotes cholesterol efflux in human blood-derived macrophages, we measured HDL- and apoAI-mediated cholesterol efflux in these macrophages. At 5 and 10 μmol/L, T0901317 significantly increased HDL-mediated cholesterol efflux by 36% (from 30.3% to 41.2%) and 50% (from 30.3% to 45.6%) (Figure 1A) as well as apoA-I-mediated cholesterol efflux by 115% 9 (from 2.6% to 5.6%) and 165% (from 2.6% to 6.9%) (Figure 1B), respectively.

### LXRs agonist T0901317 increases mRNA and protein expression levels of genes involved in cholesterol efflux in human macrophages

**Figure 2:**
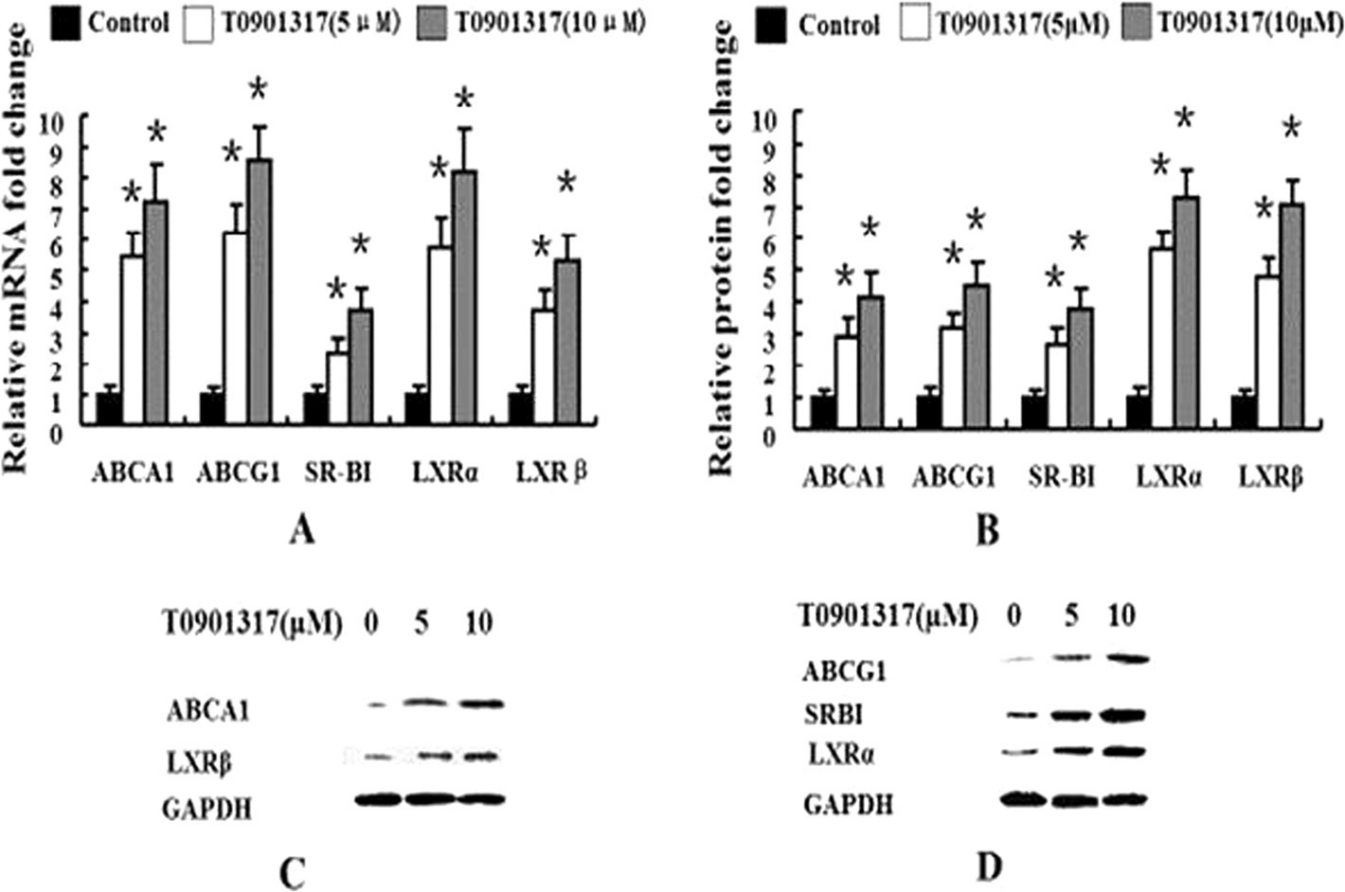
**LXR agonist T901317 increases expression of genes related to cholesterol efflux.** Human macrophages were treated without or with LXRs agonist T0901317 at 5 μmol/L or 10 μmol/L for 24 h, and ABCA1, ABCG1, SR-BI, LXRα and LXRβ mRNA expression levels were measured by real-time quantitative PCR **(A)**. Human macrophages were treated without or with LXRs agonist T0901317 at 5 μmol/L or 10 μmol/L for 48 h, and ABCA1, ABCG1, SR-BI, LXRα and LXRβ protein expression levels were measured by immunoblotting **(B, C and D)**. Similar results were obtained in three independent experiments. Data are presented as Mean ± SEM, *P < 0.05 (vs control).

ABCA1 and ABCG1 are LXR target genes critically involved in cholesterol efflux [14]. As expected, LXRs agonist T0901317 treatment increased mRNA levels of ABCA1 and ABCG1 by 550% and 605% respectively at 5 μmol/L, and 780% and 945% respectively at 10 μmol/L in human macrophages (Figure 2A). The mRNA levels of SR-BI, LXRα and LXRβ were also elevated significantly by 255%, 560%, and 365%, respectively, at 5 μmol/L, and by 470%, 895% and 515%, respectively, at 10 μmol/L. Similar changes were found for the protein levels of these genes (Figure 2B, 2C and 2D). In human macrophages, T0901317 treatment increased the protein expression levels of ABCA1, ABCG1, SR-B1, LXRα and LXRβ by 295%, 309%, 355%, 550%, and 485%, respectively, at 5 μmol/L, and by 560%, 490%, 520%, 740%, and 690%, respectively.

### LXRα rather than LXRβ sustains the baseline levels of ABCA1, ABCG1 and SR-BI expression in human macrophages

**Figure 3:**
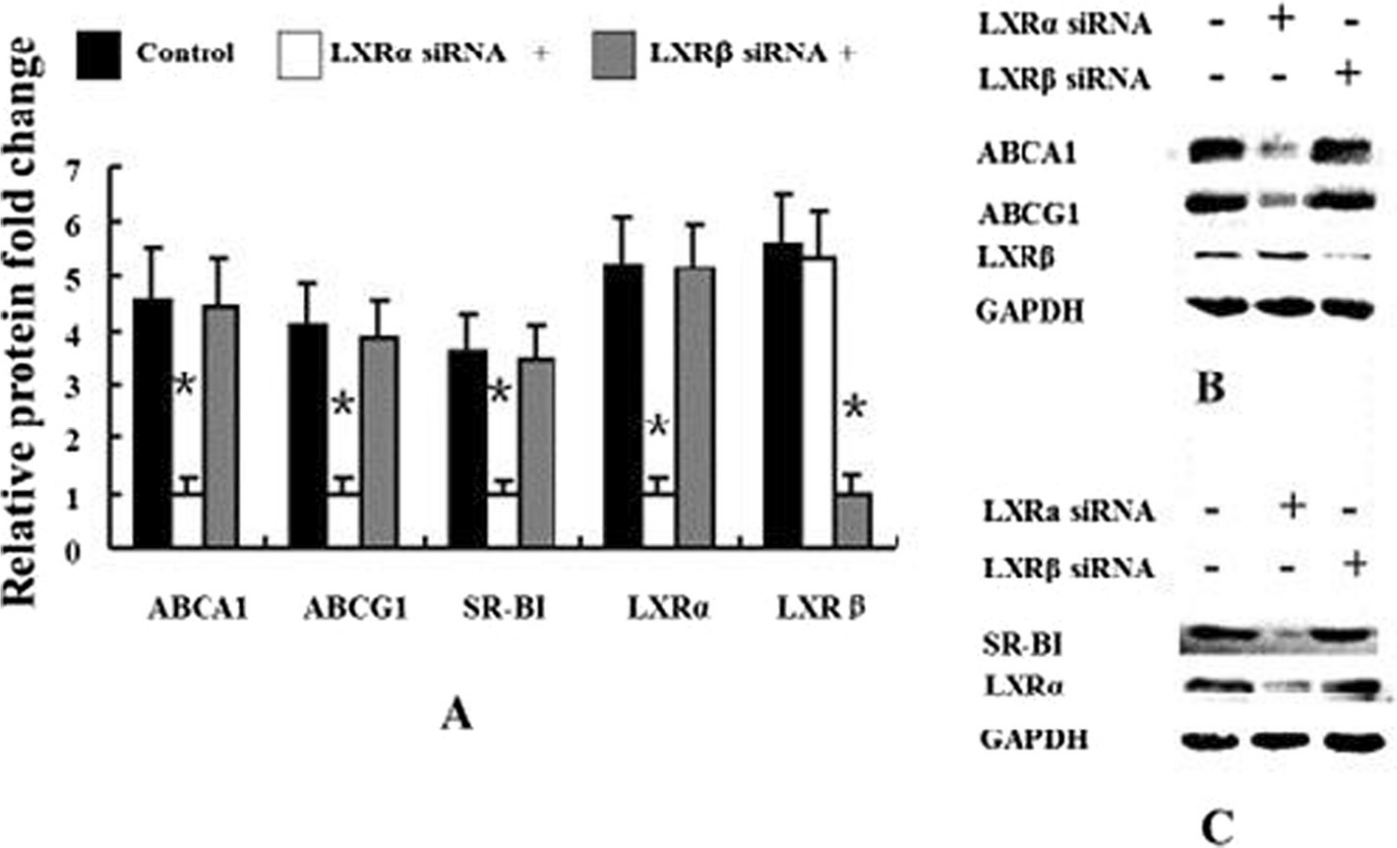
**The baseline protein expression levels of ABCA1, ABCG1 and SR-BI depend on LXRα, but not LXRβ in human macrophages.** Human macrophages were transfected with non-targeting control, LXRα siRNA, or LXRβ siRNA, and then incubated for 96 h. ABCA1, ABCG1, SR-BI, LXRα and LXRβ protein expression levels were measured by immunoblotting **(A, B and C)**. Similar results were obtained in three independent experiments. Data are presented as Mean ± SEM, *P < 0.05 (vs non-targeting control).

To determine the relative importance of each LXR isoform in maintaining baseline levels of ABCA1, ABCG1 and SR-BI expression in human macrophages, we independently silenced each LXR isoform in these cells. Silencing of LXRα (~79% efficiency) in human macrophages reduced the protein expression levels of ABCA1, ABCG1, and SR-BI to 21.7%, 24.4%, and 28.2% of each non-targetin control siRNA group, respectively, without affecting the protein expression of LXRβ. However, when LXRβ expression was silenced by ~78% in human macrophages, the protein expression levels of ABCA1, ABCG1, and SR-BI were not affected (Figure 3A, 3B and 3C).

### LXRα rather than LXRβ sustains the baseline cholesterol efflux in human macrophages

**Figure 4:**
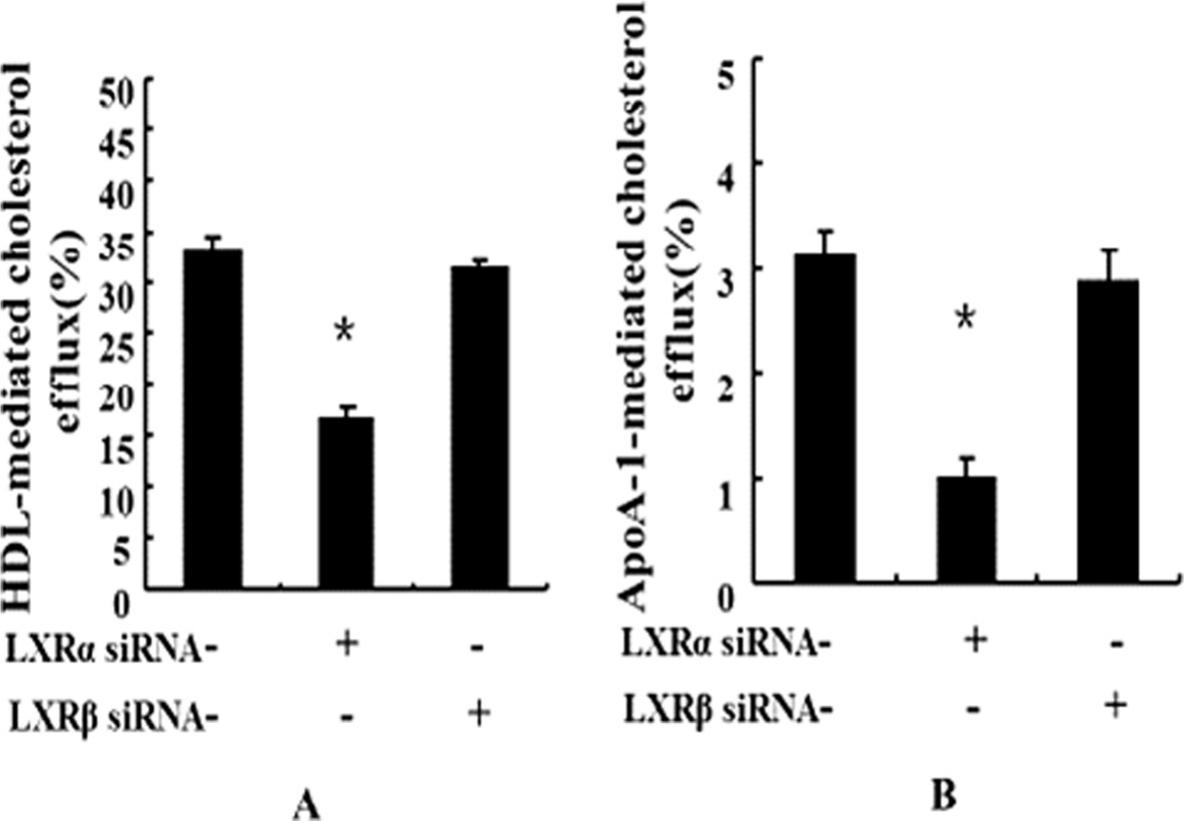
**The baseline levels of cholesterol efflux depend on LXRα, but not LXRβ. in human macrophages.** After labeled with [^3^H]-cholesterol for 24 h, human macrophages were transfected with non-targeting control, LXRα , or LXRβ siRNA, and incubated in DMEM containing 0.2% BSA for 72 h. The media were then replaced with DMEM containing 0.2% BSA in the presence of apoA-I (10 μg/mL) **(A)** or HDL (50 μg/mL) **(B)** for 24 h, a ssayed for cholesterol efflux in triplicate as described under the Methods. Data are presented as Mean ± SEM, *P < 0.05 (vs non-targeting control).

To determine the relative contribution of each LXR isoform to baseline levels of cholesterol efflux in human macrophages, we independently silenced each LXR isoform by the siRNA approach and measured HDL- and apoA-I-mediated cholesterol efflux in these cells (Figure 4A and 4B). LXRα siRNA treatment significantly reduced HDL-, and apoA-I- dependent cholesterol efflux by ~48% (from 33% to 17.2%) and ~69% (from 3.1% to 0.95%), respectively, in human macrophages. However, these effects were not observed in human macrophages treated with LXRβ siRNA.

## Discussion

The major finding of this study is that LXRα rather than LXRβ expression is required for sustaining the baseline protein expression levels of ABCA1, ABCG1 and SR-BI, as well as HDL- and apoA-I- mediated cholesterol efflux in human blood-derived macrophages. There has been increasing interest in developing LXR agonists to promote cholesterol efflux and subsequent reverse cholesterol transport. LXRα activation was shown to promote cholesterol efflux and reverse cholesterol transport [15], making LXRs as attractive drug targets. However, simultaneous activation of both LXRα and LXRβ by a synthetic agonist T0901317 induces hepatic steatosis [16], an unwanted side effect. It was speculated that compounds activating LXRα in a tissue-specific manner or specifically targeting LXRα might be useful. Results from subsequent animal studies are in agreement with this speculation [17,18]. Our results demonstrate that the role of LXRα in mediating cholesterol efflux in human macrophages is minimal. While developing LXR agonists for use in humans, we may need to focus on agonists specifically targeting LXRα isoform. Our data are consistent with previous findings showing that LXRs agonist T0901317 increases expression of ABCA1 and ABCG1 to mediate cholesterol efflux in macrophages [13]. SR-BI mediates bidirectional cholesterol flux [19]. Here we found that T0901317 increases SR-BI mRNA and protein levels in human blood-derived macrophages. It is currently unclear how LXRs agonist T0901317 regulates SR-BI mRNA and protein expression. Nonetheless, increased SR-BI expression has the potential to increase the bidirectional cholesterol transport, thus unlikely contributing to increased cholesterol efflux in T0901317-treated human macrophages. The relative importance of each LXR isoform in sustaining baseline expression levels of ABCA1 and ABCG1 as well as cholesterol efflux was unclear in human macrophages. In this study, we found that LXRα isoform is essential and LXRβ is dispensable for maintaining baseline ABCA1 and ABCG1 expression and cholesterol efflux in human blood-derived macrophages. During the preparation of this manuscript, a study with similar results was published [20], demonstrating that our finding is a reproducible phenomenon in human macrophages. The two studies collectively warrant detailed molecular studies of this reproducible observation in the future.

## Conclusion

In conclusions, LXRα rather than LXRβ plays a predominant role in mediating cholesterol efflux in human macrophages.

## Competing interests

The authors declare that they have no competing interests.

## Authors’ contributions

AZSM designed the experiments and wrote the manuscript; AZSM carried out cell culture, the molecular genetic studies, the immunoassays and inhibition using LXR siRNA. ZYS participated in study design andcoordination and helped to draft the manuscript. All authors read and approved the final manuscript. QZ performed cellular cholesterol efflux experiments and statistical analyses.

## Pre-publication history

The pre-publication history for this paper can be accessed here:

http://www.biomedcentral.com/1471-2261/14/80/prepub
